# Prevalence of Mood Disorders and Associated Factors at the Time of the COVID-19 Pandemic: Potocol for a Community Survey in La Manouba Governorate, Tunisia

**DOI:** 10.2174/17450179-v18-e221026-2022-19

**Published:** 2022-11-02

**Authors:** Uta Ouali, Amina Aissa, Salsabil Rjaibi, Nada Zoghlami, Yosra Zgueb, Amine Larnaout, Mejdi Zid, Imen Kacem, Fatma Charfi, Maria Francesca Moro, Nadia Touihri, Wahid Melki, Hajer Aounallah-Skhiri, Fethi Nacef, Riadh Gouider, Zouhaier El Hechmi, Mauro Giovanni Carta

**Affiliations:** 1 Department Psychiatry A, Razi Hospital La Manouba, Tunis 1068, Tunisia; 2 Faculty of Medicine of Tunis, University of Tunis El Manar, Tunis 1068, Tunisia; 3 Research Laboratory LR18SP03, Tunisia; 4 National Institute of Health, Tunis, Tunisia; 5 Department Psychiatry D, Razi Hospital La Manouba, Manouba 2010, Tunisia; 6 Department of Neurology, Razi Hospital La Manouba, Manouba 2010, Tunisia; 7 Department of Child Psychiatry, Mongi Slim Hospital La Marsa, Marsa, Tunisia; 8 Mailman School of Public Health, Columbia University, NY, US; 9 National Institute of Statistics, Tunis, Tunisia; 10 Technical Committee for Mental Health Promotion at the Ministry of Health, Tunis, Tunisia; 11 Laboratoire de Recherche en Epidémiologie Nutritionnelle (SURVEN), Tunis, Tunisia; 12 Private Practice, Tunis, Tunisia; 13 Center for Liaison Psychiatry and Psychosomatics, University Hospital, University of Cagliari, Cagliari, Italy

**Keywords:** Study protocol, Community survey, Prevalence, Mood disorder, Depression, Bipolar disorder, COVID-19

## Abstract

**Aims:**

The present survey aims to assess the overall mood disorder prevalence and identify associated socio-demographic and clinical factors in a Tunisian community sample, with special attention to the COVID-19 pandemic.

**Background:**

Mood disorders are one of the leading causes of all non-fatal burdens of disease, with depression being at the top of the list. The COVID-19 pandemic may have increased the prevalence of mood disorders, especially in Low and Middle-income countries (LMICs) and in vulnerable populations.

**Objective:**

1/ Assess point and lifetime prevalence of depressive and bipolar disorders as well as subthreshold bipolarity in a representative population sample of La Manouba governorate and assess treatment patterns for these disorders; 2/Study socio-demographic and clinical correlates of mood disorders 3/ Assess the association between mood disorders and quality of life 4/ Study the impact of the COVID-pandemic on the prevalence of mood disorders 5/ Assess coping mechanisms to the COVID-pandemic and whether these mechanisms moderate the appearance of mood disorders or symptoms since the beginning of the pandemic

**Methods:**

This is a household cross-sectional observational survey to be conducted in La Manouba Governorate in a sample of 4540 randomly selected individuals aged ≥ 15 years. Data collection will be carried out by trained interviewers with clinical experience, through face-to-face interviews and the use of the computer assisted personal interviewing approach (CAPI). The following assessment tools are administered:

**Results:**

Structured clinical Interview for DSM IV-TR (Mood disorder section and Screening questions on Anxiety), Mood Disorder Questionnaire (MDQ), Suicide Behaviors Questionnaire-Revised (SBQ), 12-item Short Form Survey (SF-12), the Brief-COPE, and a questionnaire about a headache. In addition, socio-demographic and clinical data will be collected.

**Conclusion:**

This will be one of the very few household surveys in a general population sample to assess mental health problems and COVID-19-related variables since the beginning of the pandemic. Through this research, we aim to obtain an epidemiological profile of mood disorders in Tunisia and an estimation of the impact of the COVID-19 pandemic on their prevalence. Results should contribute to improving mental health care in Tunisia.

## 1 INTRODUCTION

The COVID-19 pandemic, caused by SARS-CoV-2, has dramatically changed the way we live globally over the past two years. COVID-19 exploded in December 2019 in Wuhan city, China, and almost immediately, given the speed of propagation, in January 2020, the WHO declared it a public health emergency of international concern [1]. By December 2021, COVID-19 had infected around 280 million people and caused more than 5.300.000 mortalities worldwide [2].

The pandemic is suspected to have dramatically increased the prevalence of mood disorders [3, 4], and as a consequence increased suicidality [5]. It is also believed that the impact may be greater in the most vulnerable and poor sections of the population [4, 6]. This estimated increase may be determined by various factors such as the fear of getting sick [7], the social isolation due to lockdown [8, 9], the severe economic impact [10], the death of family and friends [11], and the long-term consequences of Covid disease (Long Covid) [12].

However, although numerous studies on the subject have been conducted through questionnaires via the internet or telephone [10, 13], or with direct interviews but only in specific populations, i.e. on health workers [3], general population household surveys on the subject are rare to non-existent. However, general population surveys conducted door to door are obviously more suitable for defining precise prevalence rates as well as for studying factors associated with morbidity and resilience, as they are representative of the general population and not affected by selection biases such as internet familiarity. This could exclude precisely the most economically vulnerable people and therefore, probably, the most at risk.

Mental and substance use disorders are the leading global cause of all non-fatal burdens of disease, accounting for 21,2% of years lived with disability (YLDs) overall [14]. However, according to other authoritative sources, this figure could be even underestimated and the true global burden of mental illness might in fact account for 32.4% of YLDs [15].

Major Depressive Disorder is the mental health diagnosis with the greatest impact, accounting for 40.5% of the total DALYs due to mental and substance use disorders [16]. Indeed, depression is one of the most frequent mental disorders, and cross-national studies of major depression have shown lifetime prevalence rates of about 11 – 15% [17] and an annual prevalence of 4.6% [18].

Bipolar disorders, with lifetime prevalence rates between 1% to 2.4% being relatively rare compared to depressive disorders, are particularly disabling due to their early onset, severity and chronicity, and account for 7% of all DALY’s due to mental and substance use disorders [16, 19, 20]. However, current diagnostic methods in epidemiological surveys such as employing lay interviewers and highly structured interviews may underestimate their prevalence [21].

Mood disorders have high rates of somatic and psychiatric comorbidity and have a huge impact on the social and professional life of people, in addition to their impact on patient's family members, friends, and colleagues [20]. One of the major complications of all mood disorders is suicidality. Indeed, about one-half to two-thirds of all suicides are committed by people who suffer from mood disorders [22]. In addition, across both high- and low- and middle-income countries, females are disproportionately affected by mood disorders in terms of prevalence rates as well as disability [14].

Geographic variations exist within worldwide prevalence and DALY rates of mental disorders. One study shows, that mental disorders DALYs are highest in North Africa and the Middle Eastern regions, which, according to the authors, could partially be attributed to the high number of wars and conflicts within these regions [14, 23]. However, there is a shortage of mental health related data and research from the Arab world in general and on prevalence rates and correlates of mood disorders in particular [24, 25].

In Tunisia, the only epidemiological study on mental disorders on a representative sample of the general population of Ariana governorate (n=5000) was conducted in 1995 [26]. Ariana governorate was chosen, as it came closest to the socio-demographic characteristics of the general population of Tunisia at this time. The study has revealed lifetime prevalences of depressive disorders and psychotic disorders of 8% and 1%, respectively. Bipolar disorder was not investigated in this study. Less than 10% of the study participants that were diagnosed with depressive disorder received appropriate treatment. This is in line with international studies showing that only a minority of patients with Major depression received minimally adequate treatment: 1 in 27 in low-/lower-middle-income countries [18].

Since 1995, Tunisia has undergone deep political and socio-economic changes, which seemed to have a significant impact on the population mental health. One study showed that following the Tunisian revolution in 2011, psychiatric outpatient consultations received a higher number of patients with a diagnosis of major depressive disorder, adjustment disorder, and post-traumatic stress disorder [27]. Another study found that within 8 years before and after the Tunisian revolution, the number of suicides rose 1.7 times, with the prevalence rising from 1.8 to 3.12 suicides per 100,000 persons per year [28].

Rising awareness of the frequency of mental health problems in Tunisia led the investigators of the « Tunisian Health Examination Survey » (THES) to include some questions on mental health. This study was organized by the WHO and the National Institute of Public Health of Tunisia (NIH), and conducted in 2016 on a nationally representative sample of 9209 participants aged 15 years and above. Results showed lifetime prevalence rates of diagnosed and treated depression of 4.7% (4% in males, and 5,4% in females) and suicidal ideation among those having at least one depressive symptom being 10.1% [29]. However, the study did not evaluate the prevalence of depression according to full diagnostic criteria nor did it evaluate other types of mood disorders.

Tunisia has been heavily impacted by the COVID-19 pandemic. Indeed, as of the end of December 2021, there have been more than 700,000 confirmed cases of COVID-19 with more than 25,000 deaths in Tunisia, which is one of the highest mortality rates on the African continent and in the WHO-EMRO region [30].

Besides the high morbidity and mortality rate, the COVID-19 pandemic acted in Tunisia, more than in many high-income countries, as a socio-economic stressor. Insufficient social and health insurance, a high percentage of the population working in the informal sector, and a very difficult pre-pandemic economic and financial situation affecting large parts of the population, significantly added to the stress of the virus itself.

The lack of epidemiological data on mental health in Tunisia and the under-detection of mood disorders have brought into focus the need to conduct regular epidemiological surveys in the mental health field. In addition, given the high morbidity rate of COVID-19 in Tunisia, its impact on the mental health of the Tunisian population would need a more thorough assessment.

The present survey aims therefore to assess the overall mood disorder prevalence and identify associated socio-demographic and clinical factors in a Tunisian community sample, with special attention to the COVID-19 pandemic. The specific aims are to:

1) Assess point and lifetime prevalence of depressive and bipolar disorders as well as subthreshold bipolarity in a representative population sample of La Manouba governorate and assess treatment patterns for these disorders;

2) Study socio-demographic and clinical correlates of mood disorders

3) Assess the association between mood disorders and quality of life

4) Study the impact of the COVID-pandemic on the prevalence of mood disorders

5) Assess coping mechanisms to the COVID-pandemic and whether these mechanisms moderate the appearance of mood disorders or symptoms since the beginning of the pandemic

## 2 MATERIALS AND METHODS

### 2.1 Study Design

This is a cross-sectional, household, descriptive, observational study (“Community survey”).

### 2.2 Setting

This household survey will be conducted in La Manouba governorate, which is one of the twenty-four governorates in Tunisia and is situated in inland, northern Tunisia. One part of this governorate belongs to the Greater metropolitan area of Tunis. La Manouba Governorate has about 380.000 inhabitants and is characterized by demographic and socio-economic determinants comparable to the total Tunisian population according to the 2014 general census data [31]. Data collection will be performed through face-to-face interviews.

### 2.3 Participants

The survey will be conducted among a random sample of La Manouba governorate population, according to the following inclusion criteria: age≥ 15 years, being a resident of La Manouba governorate, capable of understanding the Arabic language, having sufficient cognitive capacities to understand the interview questions, and having provided written informed consent, including the consent of their legal representative for those < 18 years. Not included are inhabitants of residences such as military camps, prisons, or hospitals, and individuals residing only temporarily in a household. Individuals who do not accomplish the socio-demographic and mood disorder questions of the survey will be excluded.

### 2.4 Sample Size and Sampling Method

The sample size (N) was calculated using the formula for simple random sampling:

N = ([(Z α/2)2 x P (1 - P)] / d2), and taking into consideration:

- A prevalence (P) of bipolar disorders equal to 1,6% (based on results of international prevalence studies, see Merikangas *et al*, 2012),

- An accuracy (d) equal to P/4,

- An α error risk equal to 5% (Z α/2 =1.96),

- A non-response rate equal to 20%,

According to these calculations, the required sample size would be around 4540 individuals.

Based on a 3-stages sampling design, the random sampling process was as follows: (1) 152 districts were randomly selected among all districts of La Manouba Governorate, based on 2014 Census data [31]. (2) In each district, 15 households will be randomly selected. (3) In each household, two individuals aged 15 and over (one man and one woman) will be randomly selected to be part of this study, after giving their informed consent. The sampling procedure has been carried out by the Tunisian National Institute of Statistics (INS).

### 2.5 Measures

The following standardized assessment tools will be used in this study:

#### 2.5.1 Structured Clinical Interview for DSM IV-TR (SCID-I for DSM-IV-TR)

To assess the prevalence of depressive and bipolar disorders, we will use the Mood Disorder Section of the Structured Clinical Interview (SCID-I) for DSM-IV-TR [32]. The SCID is a clinician-administered structured clinical interview using an algorithm to evaluate the principal diagnoses of the DSM. We will use the Arabic version of the SCID modified for the World Mental Health Survey reappraisal interviews [33] with the following adaptations: (i) mixed features of a manic, hypomanic or depressive episode will be evaluated according to DSM-5, (ii) subthreshold bipolar symptoms: brief hypomanic episodes of ≥2 and ≤4 days, and hypomanic episodes with insufficient symptoms according to DSM-5 criteria. To assess the impact of the COVID-pandemic on mood disorder prevalence, we will record not only point-prevalence and lifetime-prevalence of depressive and bipolar disorder, but also its prevalence since the beginning of the pandemic in Tunisia (the first confirmed case was recorded in Tunisia on March 4, 2020) [34]. We will also record if the appearance of a mood episode was the direct effect of a COVID infection.

To detect signs and symptoms of anxiety disorders, we will include the Anxiety Screening questions of the SCID-I for DSM-IV-TR concerning panic attacks, agoraphobia, social phobia, specific phobias and generalized anxiety [32].

### 2.6 Mood Disorder Questionnaire (MDQ)

The Mood Disorder Questionnaire (MDQ) will be used to assess (subthreshold) bipolarity and to confront it with SCID diagnoses of depression, bipolar disorder and subthreshold bipolar signs and symptoms (short-duration hypomania and hypomania with insufficient symptoms). The MDQ was developed as a screening instrument for bipolar disorder and consists of 15 items; 13 yes/no items concerning mood signs and symptoms and two questions about symptom co-occurrence and impact on functioning [35]. It has been widely used in clinical and non-clinical populations of different cultures, and its validity as a screening instrument has been well established [36]. In our study, we will use the version adapted to Standard Arabic and validated in a clinical sample of 151 Tunisian patients with mood disorders [37].

### 2.7 Suicide Behaviors Questionnaire-revised

To assess suicidality, the Suicide Behaviors Questionnaire-revised (SBQ-R) will be administered [38]. It has 4 items each exploring a different dimension of suicidality: 1/ lifetime suicidality, 2/frequency of suicidal ideation over the past 12 months, 3/ threat of suicide attempt, and 4/ likelihood of future suicidal behavior. The original version of the SBQ-R was previously adapted to Standard Arabic and used in a population of 821 Tunisian High School students [39].

### 2.8 12-item Short Form Survey (SF-12)

To assess health-related quality of life, we will use the 12-item- Short Form Health [40]. This is a self-report instrument that has been derived from the 36 – item Short Form Survey [41]. The SF-12 is widely used in general population studies since it is practical, reliable and valid, and produces similar results for physical and mental health scores with far less respondent burden [42]. It assesses eight health domains through one or two questions per domain: Physical Functioning, Role-Physical, Bodily Pain, General Health, Vitality, Social Functioning, Role-Emotional, and Mental Health. Each health domain score contributes to the Physical Component Summary (PCS) and Mental Component Summary (MCS) scores. In Tunisia, the original 36-item version of the questionnaire has been validated in Standard Arabic [43]. For our study, we will retrieve the 12 items used for SF-12 from the scale validated in Standard Arabic.

### 2.9 Brief-COPE

To determine the coping strategies towards the COVID-19 pandemic used by the Tunisian general population, we will administer the Brief-COPE. This is a 28-item auto-questionnaire assessing adaptive and nonadaptive coping-strategies; 14 coping strategies are covered by 1 item each [44]. It is the most widely validated coping questionnaire worldwide [45]. Several attempts have been made to shorten the inventory, but the factor structure remains debated [45]. For the purpose of our study, the 28-item original version was adapted to Arabic and piloted in 40 individuals from the general population. According to results from pilot testing and expert opinion, the language of the questionnaire was further refined and the number of items was then reduced to 14, each item pertaining to one strategy.

### 2.10 Questionnaire about Headache

Migraine is known to have high comorbidity with mood disorders, especially bipolar disorder [46]. To screen for migraine, we established a 5-item-screening-questionnaire based on the Migraine-4 questionnaire, an algorithm developed to screen for migraine in the general population [47]. The newly-established questionnaire covers the most frequent signs and symptoms of migraine and was translated into Tunisian Arabic.

### 2.11 Socio-demographic and Clinical Variables

Socio-demographic variables will include Age, gender, marital status, level of education, professional situation, socio-economic status, and history of migration.

Clinical variables will include items related to (i) Access to care for mental health problems, including non-medical (traditional healers), general medical, and specialist care; (ii) COVID: history and eventual severity of COVID infection, vaccination status, anxiety due to contracting COVID, (iii) personal history of a medical disorder (diabetes, hypertension, other chronic illness), and (iv) problems related to addiction within the family and access to care for addiction.

### 2.12 Procedures

#### 2.12.1 Data Collection

Data collection will be carried out by trained interviewers through face-to-face structured individual interviews with an estimated duration of the interview of 45 min - 1 h per participant.

All 26 interviewers carrying out the fieldwork will be selected amongst interns in medicine and residents in family medicine as well as psychology students having graduated from their master studies. The selected interviewers will receive a full 4-day training workshop, during which they are familiarized with general and ethical aspects of data collection, the signs and symptoms of mood disorders, the use of the SCID algorithm, the conduct of interviews through roleplays, and the use of the electronic tablet.

Using the computer-assisted personal interviewing approach (CAPI), most of the required checks for inconsistent and missing data will be integrated into the system. This gives the interviewer the opportunity to correct inconsistent data as they occur, and repeat the question if necessary, to get a better answer quality.

A pilot study will be conducted allowing for adjustments to the interview questions and of the data collection process in the field site.

Regular supervision of interviewers will be ensured through: (i) a one-day workshop after the pilot phase will allow interviewers to express any difficulties encountered during fieldwork, (ii) repeated checks on the field site, and (iii) regular telephone contact with the interviewers. Each interviewer will be assigned a supervisor at the beginning of the pilot study.

#### 2.12.2 Data Management Plan

Data entry will be directly performed via tablets during the interview using the Android version of CSPRO software: the Cspro (6.2) application. All data will be centralized in the National Institute of Health (NIH), where cleaning, coding and analysis will be performed with R software.

Qualitative variables will be described by simple counts and percentages presented with 95% confidence intervals (95% CI), and quantitative variables by mean ± SEM (standard error of mean). The association between mood disorders (dependent variables) and socio-economic and clinical data (explanatory variables) will be first examined in univariate analysis using the chi-squared test. Brut Odds Ratios (ORs) with 95% CI will be used to quantify this association. The multivariable analysis will then be performed using binary logistic regression to assess the association between mood disorders and explanatory variables while adjusting for confounding factors and effect modification if needed. The model building will be done using backward procedures. Only variables that retained statistically significant associations with the dependent variable will be considered and will be presented with Adjusted Odds Ratios (AORs) and 95% CI. A p-value< 5% will be considered statistically significant.

### 2.13 Ethical Considerations

The study protocol was approved by the ethics committee of Razi University Hospital, the Research Directorate of the Tunisian Ministry of Health, the Regional Directorate of Health of La Manouba Governorate, the National Instance of Protection of Personal Data, and the National Council of Statistics.

Before study entry, informed consent will be obtained from every enrolled participant or from the legal representative for participants aged less than 18 years or participants otherwise unable to give consent (*e.g*. participants with severe mental illness).

The survey does not involve any risk to the physical health of the study participants. However, study participants might feel discomfort induced by some of the interviewer's questions or the length of the interview. Interviewers will be trained during the workshop on how to approach potential study participants, and how to respond if a participant shows signs of discomfort.

In case a psychiatric disorder or a psychiatric emergency (especially acute suicidality) is detected in one of the study participants, an appropriate treatment will be proposed.

Fieldworkers will work in pairs, consisting ideally of one male and one female fieldworker. This allows for fieldworkers to interview participants of the same sex, which could help in reducing their discomfort. Moreover, it ensures additional safety for the field workers.

Confidentiality of all study participant data will be ensured. Registered cases will be gathered with an automatic unique identification code in the final database and no one will have access to participants’ names which will be kept and protected in a locked area until the end of the study and will then be deleted.

The training of interviewers will be conducted according to the COVID-19-related safety guidelines of the Tunisian Ministry of Health. During fieldwork (household surveys), social distancing, wearing of face mask and use of hand sanitizer will be mandatory for all interviewers. In addition, we ensure that all interviewers have a full vaccination status prior to starting the fieldwork.

## 3 RESULTS AND DISCUSSION

### 3.1 Strengths of the Study

To the best of our knowledge, this will be one of the rare if not the only household survey in a general population sample to assess the prevalence of mood disorders, associated mental health problems, and COVID-19-related variables since the beginning of the pandemic. Indeed, as face-to-face interviews allow the detection of visual signs, they seem to be best to maximize the quality of the data collected, especially for information considered to be sensitive, such as mental health issues [48]. A systematic review comparing the validity of diagnostic telephone interviews versus diagnostic face-to-face interviews concluded that telephone interviewing in the general population may not be valid because comparability measures are lowest in these low-risk populations, especially for low prevalence disorders such as bipolar disorder [49]. In addition, telephone surveys have been found to be less suitable for people who have impaired hearing, are older, are from minorities or a lower socio-economic class or educational level [48, 50, 51].

More so than telephone surveys, internet surveys have an inherent risk of selection bias if sample representativeness of the target population cannot be guaranteed. A systematic review of the efficiency and quality of data collection among public mental health surveys conducted during the COVID-19 pandemic included 37 relevant surveys of the general public and found that the data collection methods used were efficient but generally had a high risk of bias [52].

The second strength is the conduct of the interviews by residents in family medicine or psychologists. In Tunisia, residents in family medicine have to undergo a 3-month clinical practice training in psychiatry. This ensures that interviewers have basic clinical knowledge in psychiatry and mental health as well as clinical interviewing skills which is important for obtaining accurate SCID ratings. Indeed, these ratings require some clinical judgment with respect to requesting participants to elaborate when symptom presence or absence is unclear, and with respect to the diagnostic presence [53]. There is inconclusive evidence concerning the validity of diagnoses by interviewer type (lay interviews versus clinician interviewers): A small number of studies have found no differences, whereas a larger number of studies have found differences in prevalence estimates of disorders by interviewer type [54].

## 3.2 Challenges of the Study

The challenges are essentially related to the COVID-19 pandemic. The protocol was adapted to ensure the safety of participants and fieldworkers. All fieldworkers are required to have an up-to-date Corona vaccination status. Given the ongoing pandemic, some potential participants might refuse to participate because of fear of infection. However, the vaccination rate in Tunisia is relatively high compared to other LMICs: about 48% of the population has been fully vaccinated, and more than 55% had at least one vaccination [34].

## CONCLUSION

Through this research, we aim to obtain an epidemiological profile of mood disorders in Tunisia, and an estimation of the impact of the COVID-19 pandemic on their prevalence. Results are important to guide national public health policies in the mental health field and should contribute to improving mental health care in Tunisia.

## Data Availability

Not applicable.

## LIST OF ABBREVIATIONS

LMICs: Low and Middle income countries
CAPI.: Computer Assisted Personal Interviewing Approach
MDQ: Mood Disorder Questionnaire
SBQ: Suicide Behaviors Questionnaire-Revised
THES: Tunisian Health Examination Survey
SF-12: Short Form Survey
NIH: National Institute of Health
ORs: Odds Ratios
